# Metabolic variation in natural populations of wild yeast

**DOI:** 10.1002/ece3.1376

**Published:** 2015-01-14

**Authors:** Pedram Samani, Etienne Low-Decarie, Kyra McKelvey, Thomas Bell, Austin Burt, Vassiliki Koufopanou, Christian R Landry, Graham Bell

**Affiliations:** 1 Department of Biology, McGill UniversityMontreal, Québec, Canada, H3A 1B1; 2 School of Biological Sciences, University of EssexWivenhoe Park, Colchester, CO4 3SQ, U.K; 3 Department of Psychology, University of TorontoToronto, Ontario, Canada, M5S 3G3; 4 Department of Life Sciences, Imperial College LondonSilwood Park Campus, Ascot, Berkshire, SL5 7PY, U.K; 5 Département de Biologie, Université LavalQuébec, Québec, Canada, G1V 0A6

**Keywords:** Ecological diversification, evolution, genetic variation, metabolic trade-offs, microbial metabolic diversity, *Saccharomyces paradoxus*

## Abstract

Ecological diversification depends on the extent of genetic variation and on the pattern of covariation with respect to ecological opportunities. We investigated the pattern of utilization of carbon substrates in wild populations of budding yeast *Saccharomyces paradoxus*. All isolates grew well on a core diet of about 10 substrates, and most were also able to grow on a much larger ancillary diet comprising most of the 190 substrates we tested. There was substantial genetic variation within each population for some substrates. We found geographical variation of substrate use at continental, regional, and local scales. Isolates from Europe and North America could be distinguished on the basis of the pattern of yield across substrates. Two geographical races at the North American sites also differed in the pattern of substrate utilization. Substrate utilization patterns were also geographically correlated at local spatial scales. Pairwise genetic correlations between substrates were predominantly positive, reflecting overall variation in metabolic performance, but there was a consistent negative correlation between categories of substrates in two cases: between the core diet and the ancillary diet, and between pentose and hexose sugars. Such negative correlations in the utilization of substrate from different categories may indicate either intrinsic physiological trade-offs for the uptake and utilization of substrates from different categories, or the accumulation of conditionally neutral mutations. Divergence in substrate use accompanies genetic divergence at all spatial scales in *S. paradoxus* and may contribute to race formation and speciation.

## Introduction

Populations adapt to altered conditions of growth through natural selection, provided that there is genetic variation in fitness. Adaptation may be caused either by the sorting of standing genetic variation or by the cumulative selection of successive beneficial mutations (Barrett and Schluter 2008). The primary source of variation, and thereby the balance between sorting and cumulative selection, depends in part on the size of the population, because this will govern the number of beneficial mutations that arise during a given period of time. Very large asexual populations can adapt through mutation alone (Bell 2008). Experimental populations of unicellular microbes such as bacteria, phytoplankton, and yeasts can steadily improve when cultured in a novel environment (Kawecki et al. 2012) and may evolve new metabolic capabilities (Blount et al. 2008; Bell 2013). Conversely, lineages that have diverged ecologically over hundreds of millions of years may evolve similar phenotypes after a brief period of laboratory culture (Gravel et al. 2012). Animals and plants are larger and hence less abundant. The size of a local population is difficult to define because its limits are unclear, but the steep decline in maximum abundance with body size (Peters and Wassenberg 1983) implies that the local population of animals and plants experiencing altered conditions often comprises only a few hundred or a few thousand individuals. The rate of supply of beneficial mutations will be correspondingly low, and most evolutionary change will be based on standing variation (Barrett and Schluter 2008).

The relation between body size and abundance has led evolutionary studies of microbes to concentrate on mutation supply as a source of variation, whereas studies of animals and plants have emphasized standing variation. Microbial experimental evolution often involves the study of natural selection in populations descending from a single clone. Consequently, the quantity of standing variation for ecologically relevant traits in natural populations of microbes has rarely been systematically investigated. Indeed, before the genomics era, industrially or clinically relevant bacteria and yeasts were often identified by the range of substrates they metabolized, implying that variation within species is inconsequential (Garland and Mills 1991; McGinnis et al. 1996; Praphailong et al. 1997; Konopka et al. 1998). Detailed surveys of the filamentous fungus *Trichoderma,* however, have detected variation in substrate use among strains of the same species (Kubicek et al. 2003; Druzhinina et al. 2006; Hoyos-Carvajal et al. 2009). More recently, Deng et al. (2014) found evidence for metabolic diversification within species in microbial communities of the Baltic sea.

Metabolic variation has been documented both within and among populations of wild yeast (Schacherer et al. 2009). Geographically isolated populations of *Saccharomyces paradoxus* are genetically and phenotypically distinct (Leducq et al. 2014), whereas wild populations of its sister species, the domestic yeast *Saccharomyves cerevisiae,* are less varied, and the entire species expresses only about as much genetic variation as a single population of *S. paradoxus* (Liti et al. 2009). Within local populations, a previous survey of *S. paradoxus* found that the genetic difference between isolates was correlated with their distance apart (Koufopanou et al. 2006), although others were unable to detect such a relationship (Tsai et al. 2008). The pattern of phenotypic variation in microbial populations can now be characterized more easily and extensively than hitherto by the use of high-throughput phenotyping techniques (Konopka et al. 1998). Warringer et al. (2011) measured the growth of 86 isolates from five species of *Saccharomyces* sensu stricto yeasts and found extensive variation both between and within species with respect to almost 200 ecologically relevant conditions, including carbon substrates, nitrogen sources, nutrients, toxins, and physical factors (Warringer et al. 2011).

Natural selection will act to eliminate variation through the fixation of a metabolically superior generalist type, if such a type exists. Some degree of specialization may be actively maintained by diversifying selection, however, if there is negative genetic correlation such that the ranking of fitness of genotypes differs among substrates. This might be caused by functional interference between physiologically incompatible processes, or by the mutational degradation of inactive pathways, resulting in a “trade-off” between the utilization of substrates or sets of related substrates. Specialization will tend to evolve when genotypes compete for depletable resources (Geritz et al. 1998; Doebeli and Dieckmann 2000; Craig MacLean et al. 2005; Martin and Pfennig 2012). The range of specialized types that can be maintained by divergent selection will then depend primarily on the pattern of trade-offs between alternative substrates. Experiments with microbes have identified trade-offs between traits in as a cause of diversification (Gudelj et al. 2010), but the extent of metabolic variation and the pattern of trade-offs in natural populations of microbes remain poorly understood.

In this report, we extend the investigation of metabolic variation in microbial populations by investigating the use of carbon substrates by random isolates of wild yeast (*S. paradoxus*) from two local populations, one in North America and the other in Europe. This species is sexual, but outcrossing occurs very infrequently and populations are almost completely homozygous (Johnson et al. 2004; Tsai et al. 2008). Our enquiry was intended to characterize the potential diet of wild yeast populations, the amount of standing genetic variation in substrate utilization, and the pattern of trade-offs among substrates or groups of substrates. The presence of trade-offs in substrate utilization would indicate the potential for ecological diversification within and among natural populations.

## Materials and Methods

### Sites

Wild yeast is known to grow on oak trees (Bowlesa and Lachance, 1983; Naumov et al., 1998). We collected samples from the bark of oak trees at North American and European sites. The North American site included stands of white oak (*Quercus alba* L.) in old-growth forest in the McGill University nature reserve at Mont St-Hilaire, Quebec (referred to as “MSH”). The European site consisted of scattered oaks (*Quercus robur* L.) in parkland on the Silwood Park campus of Imperial College London (referred to as “Silwood”). The Silwood isolates had previously been characterized genetically by Johnson et al. (2004). GPS coordinates of the locations of the isolates are available with the data (electronic supplementary material).

### Isolates

Samples were collected using bark punches or putty, and yeast isolated by the selective culture procedure of Sniegowski et al. (2002). *Saccharomyces* sensu stricto yeasts were identified through PCR amplification of the ITS region and species identity confirmed through sequencing of CEN9 (Johnson et al. 2004; Bensasson et al., 2008). We chose 22 isolates of *S. paradoxus* from MSH and 23 isolates from Silwood at random from about 200 available from each site, subject to the constraint that no two isolates came from the same tree.

### Substrates

We used 96-well Biolog plates PM1 and PM2A (BIOLOG, Inc., Hayward, CA) to provide 190 carbon substrates. One well on each plate contains no carbon source.

### Survey technique

From frozen stocks, isolates were grown in YPD for two growth cycles of about eight generations each. 1.5 mL of each isolate was pelleted in an Eppendorf tube, washed twice with 0.75 mL minimal medium (yeast nitrogen base with no carbon source), resuspended in 1 mL minimal medium, and starved for 4 h. Following Biolog protocol, 0.25 mL of this culture was added to 20 mL IFY-0 medium (BIOLOG Inc.), 0.32 mL dye mix D (tetrazolium; BIOLOG Inc.), and distilled water to 25 mL. The Biolog plates were then inoculated with 0.1 mL per well of this suspension. Two replicates of each isolate were grown, plus a blank uninoculated plate. The plates were kept in incubators at 28°C. Optical density (OD) was scored on a plate reader at 590 nm after 24 h, 48 h, and 72 h of growth.

Tetrazolium measures the reducing power of the NADH supply of cells generated through catabolic pathways such as glycolysis and citric acid cycle. As it might not necessarily indicate the amount of growth on a given substrate, a supplementary experiment was conducted to validate the use of tetrazolium to detect metabolic activity and yield. Four PM1 and four PM2A plates were inoculated as above, using tetrazolium dye, while four PM1 and four PM2A plates were inoculated without using tetrazolium. These plates were incubated at 28°C, and optical density (OD) was scored on a plate reader at 590 nm after 24 h, 48 h, and 72 h of growth.

### Yield calculation

We estimated the yield in any given well as yield = (OD of inoculated well after 72 h of growth) − (OD of corresponding well on uninoculated plate). For any given isolate, the pattern of yield can be expressed in a standard fashion as the deviation of yield for a substrate from the average of yield over all substrates for that isolate: standardized yield = (yield of focal isolate on given substrate) − (mean yield of focal isolate over all substrates).

The average inoculum density over all strains was 5465 cells·mL^−1^ (SD 2462) for the Silwood survey. The average yield of an isolate over all substrates was weakly and negatively related to inoculum density (*r*^2^ = 0.15 for OD after 72 h); hence, yield was not adjusted for inoculum density.

Statistical analyses were conducted using the R language (R Development Core Team 2009). Data and analysis scripts are available in the electronic supplementary material. Pathways in which each substrate occurs were identified using KEGG: Kyoto Encyclopedia of Genes and Genomes (Ogata et al. 1999).

## Results

### Diet

**Figure 1:**
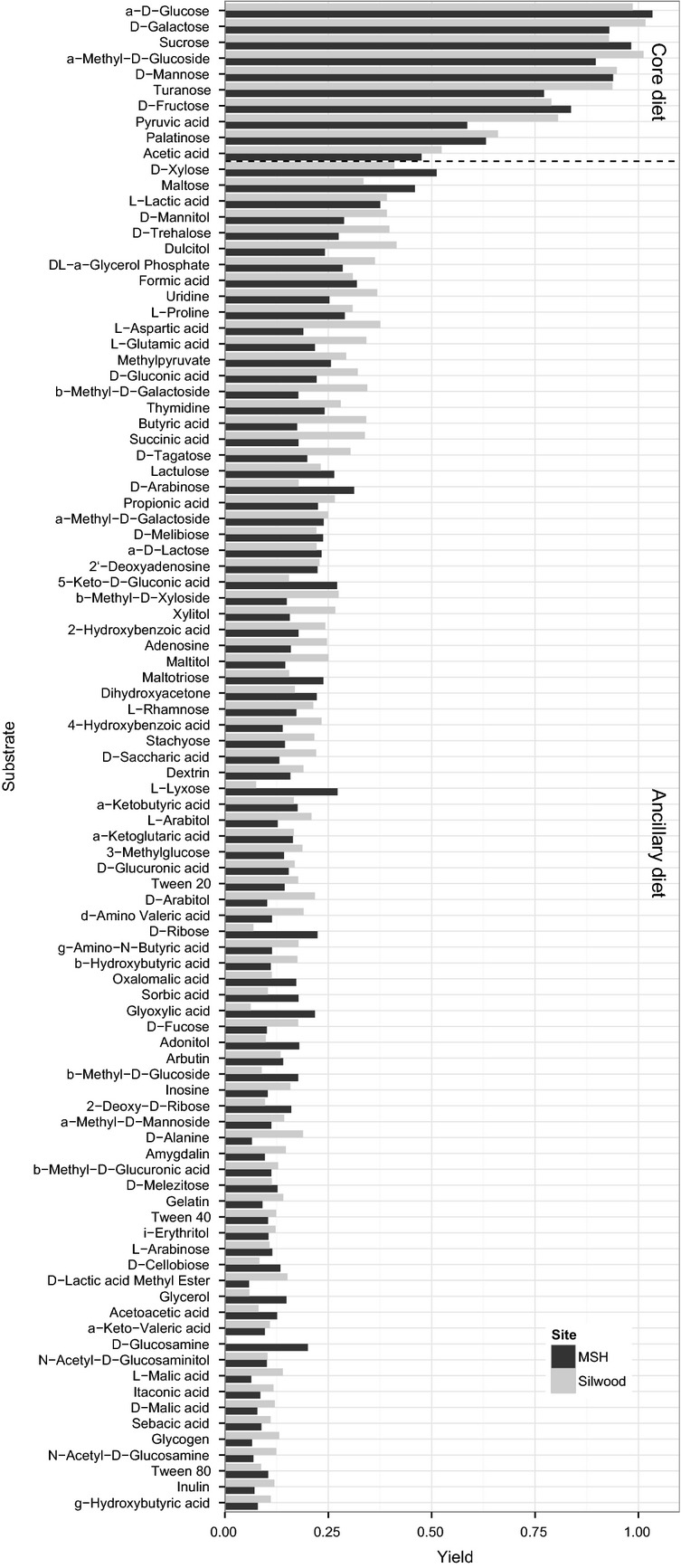
Rank distribution of yield among substrates. The bar plot shows yield for the 95 most efficiently utilized substrates in the MSH (black) and Silwood (gray) surveys. Ten substrates are clearly identified by the ability of isolates to reach higher yields on these substrates. The core diet can also be identified by the fact that all isolates in both locations achieve higher yield on these substrates (Fig.6).

We found that OD measurements with and without addition of tetrazolium were highly correlated after 72 h of growth (*R*^2^ = 0.76; Fig. S1), showing that OD accurately estimates biomass yield on a given carbon source. Almost every substrate (185/190) could be utilized by at least one isolate (*t*-test compared to value for water, testwise *P* < 0.01). The rank distribution of yield for the 50% of substrates with the highest mean scores is shown in Figure1, and these substrates are the basis of all further analysis. The other 95 substrates are very poorly utilized, giving OD readings that barely exceed the blank.

A group of 10 substrates that are used efficiently by all isolates from both sites forms a prominent shoulder at the upper end of the distribution (Fig.1). This core diet consists of the sugars glucose and fructose, their epimers (galactose, mannose), and combinations (sucrose, turanose, and palatinose). It also includes intermediary metabolites derived from these sugars (a-methyl-d-glucoside, pyruvic acid, acetic acid). The remaining substrates constitute an ancillary diet for which yield scores fall roughly linearly with increasing rank (*r*^2^ = 0.86 for linear regression of yield on rank across both sites). All MSH isolates grew well on maltose, but the Silwood isolates did not, and maltose is thus not included in the core diet.

The yield on each of these 95 substrates is highly correlated between the two surveys (*r*^2^ = 0.86). This correlation is not solely driven by the difference between core and ancillary diet; the surveys are correlated, though less strongly, even within the ancillary diet (*r*^2^ = 0.41).

### Variation among isolates

**Figure 2:**
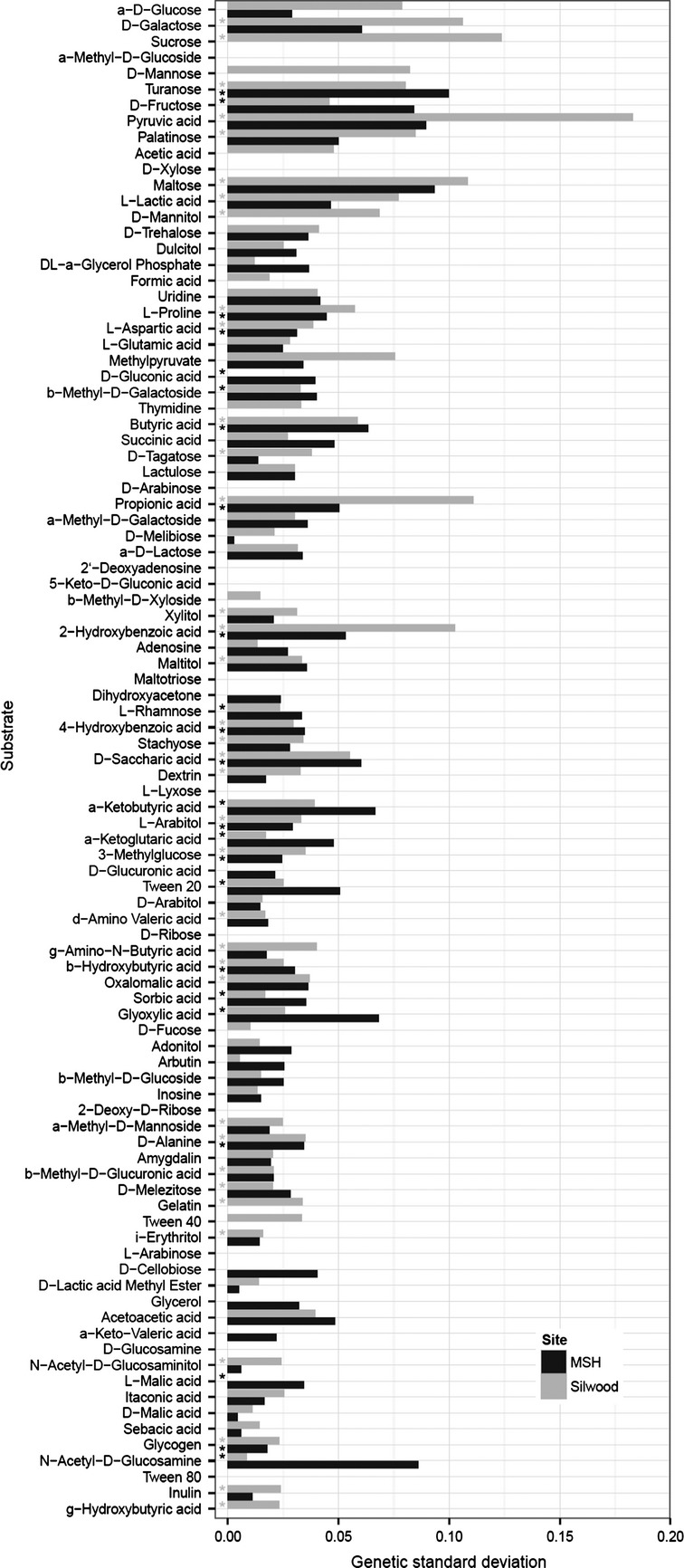
Variance of growth among substrates. The histogram bar indicates the square root of the among-isolates variance component (“genetic standard deviation”) for 72 h scores in the MSH (black) and Silwood (gray) surveys. Substrates are in the same order as in Figure1. Asterisk denotes testwise significance at *P* < 0.001 (experiment-wise *P* < 0.1).

The amount of genetic variation in yield after 72-h growth for each substrate in the core diet and the ancillary diet are shown in Figure2. The average genetic coefficient of variation over all substrates is 0.20 at MSH and 0.16 at Silwood. Isolates varied significantly (main effect of isolate, *F*-test, testwise *P* < 0.05) in yield on 13 substrates at both sites (all but turanose are from the ancillary diet); 11 substrates had significant genetic variance for MSH isolates only and 23 for Silwood isolates only. The genetic variance for given substrates is correlated between the two surveys, although less strongly than the mean (*r*^2^ = 0.17). Genetic variance is also correlated with mean yield (*r*^2^ = 0.16).

### Covariation of diet

Correlations in yield between substrates may arise even for random data, depending on the overall quantity of genetic variance (“house-car paradox” (Zuk et al. 1996)). The prevalence of positive correlations among substrates that we observed will thus be generated in part by the substantial genetic variance found in both populations. The observed distribution of pairwise correlations between substrates can be compared with the corresponding distribution obtained from randomizing the yield of each substrate among isolates. This breaks up any correlation between substrates among isolates and provides a null hypothesis with which the observations can be compared. Over all substrates, the average genetic correlation between substrates is very weakly positive (mean *r* = 0.04 with *P* < 0.001 for both MSH and Silwood sites), and the distribution does not exhibit extreme values outside the range expected from the permutation test provided by the null hypothesis. Hence, the hypothesis that growth on random substrates will be negatively correlated among random isolates is rejected by our observations. Within the whole range of substrates, however, two contrasts with substantial negative correlations could be identified.

**Figure 3:**
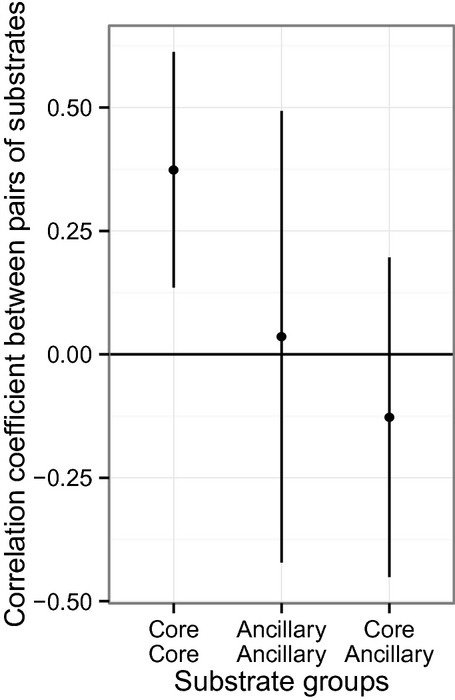
Genetic correlation coefficient between pairs of substrate grouped by diet category (mean value with standard deviation). The core/core and ancillary/ancillary groupings express correlation coefficients between pairs of substrates from the core diet and ancillary diets, respectively, whereas the core/ancillary grouping expresses correlation coefficients between a substrate from the core diet and a substrate in the ancillary diet.

The first contrast is between substrates belonging to the core and ancillary diets (Fig.3). Both within populations at each site and between isolates from different sites, yield on pairs of substrates from the same diet group (core or ancillary) is on average positively correlated (mean pairwise correlation core–core *r* = 0.37, SD = 0.24, *P* < 0.001 and ancillary–ancillary mean *r* = 0.04, SD = 0.46, *P* < 0.001), but yield on substrates from different groups is negatively correlated (mean *r* = −0.13, SD = 0.32, *P* < 0.001, test using permutation of values in one of the categories).

**Figure 4:**
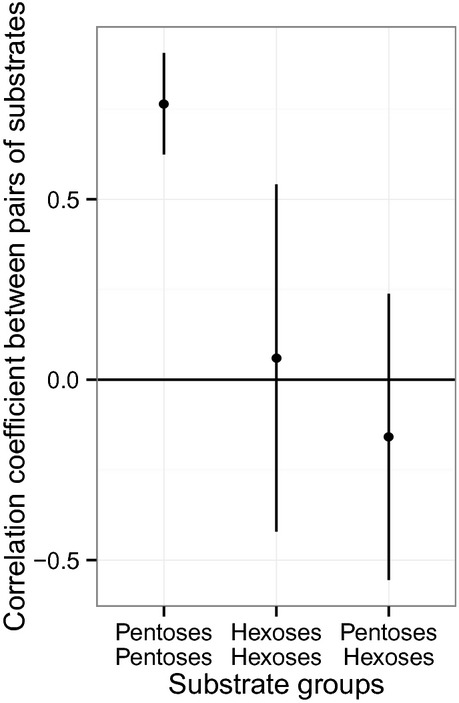
Genetic correlation coefficient between pairs of sugars grouped by number of carbon (mean value with standard deviation). Pentoses and hexoses are compared together (two values at left) or with one another (value at right).

The second contrast is between pentose and hexose sugars (Fig.4). Yield on sugars with the same number of carbon atoms is positively correlated (pentose–pentose mean *r* = 0.77, SD = 0.14, *P* < 0.001, hexose–hexose mean *r* = 0.06, SD = 0.48, *P* < 0.1); whereas yield on sugars with different numbers of carbon atoms is negatively correlated (pentose–hexose mean *r* = −0.16, SD = 0.40, *P* < 0.005).

### Continental dietary variation

**Figure 5:**
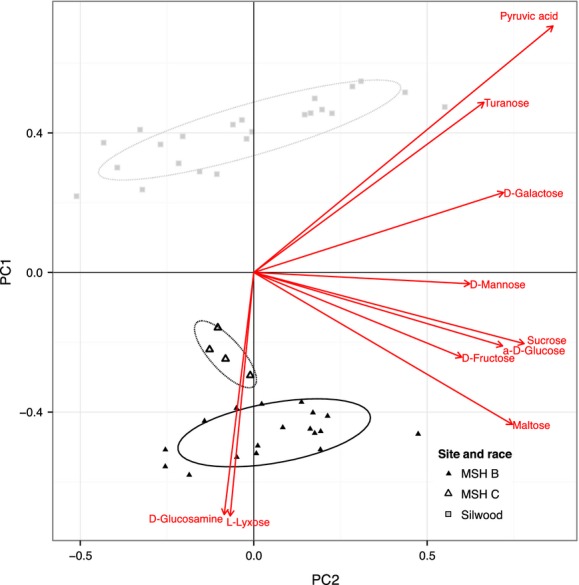
The divergence of yield among isolates summarized by projection on the two dominant principle component axes. Mean value for each isolate is presented. MSH isolates are shown as triangles, race B as solid triangles, and race C as hollow triangles, and Silwood isolates by gray squares. Ellipses show 95% confidence intervals. The ordination of the ten substrates that contribute most to PC1 are shown as red lines.

Isolates from the two localities have different patterns of substrate utilization (effect of location in ANOVA on principle component 1, *P* < 0.001, 100% proper classification when using classification algorithms such as linear discriminant analysis, Fig.5) and in this sense form distinct ecotypes. The sites are differentiated most strongly along PC1, whereas isolates within a site are differentiated along PC2.

Some substrates have consistently higher yield at one of the two site (Figs.1, 5). When grouping by substrate type, North American strains have consistently higher yield than European strains on substrates in the pentose phosphate pathway, such as 2-deoxy-d-ribose, d-arabinose, l-arabinose, d-ribose, d-xylose, and l-lyxose. Nevertheless, this is not accompanied by a consistent increase in their capacity to metabolize other substrates found in the pentose and glucuronate interconversion pathway (l-arabitol, d-arabitol, and xylitol lead to lower yield in North American strains). This difference in the capacity to utilize pentoses appears to be one of the main differences between the sites (Fig.5).

**Figure 6:**
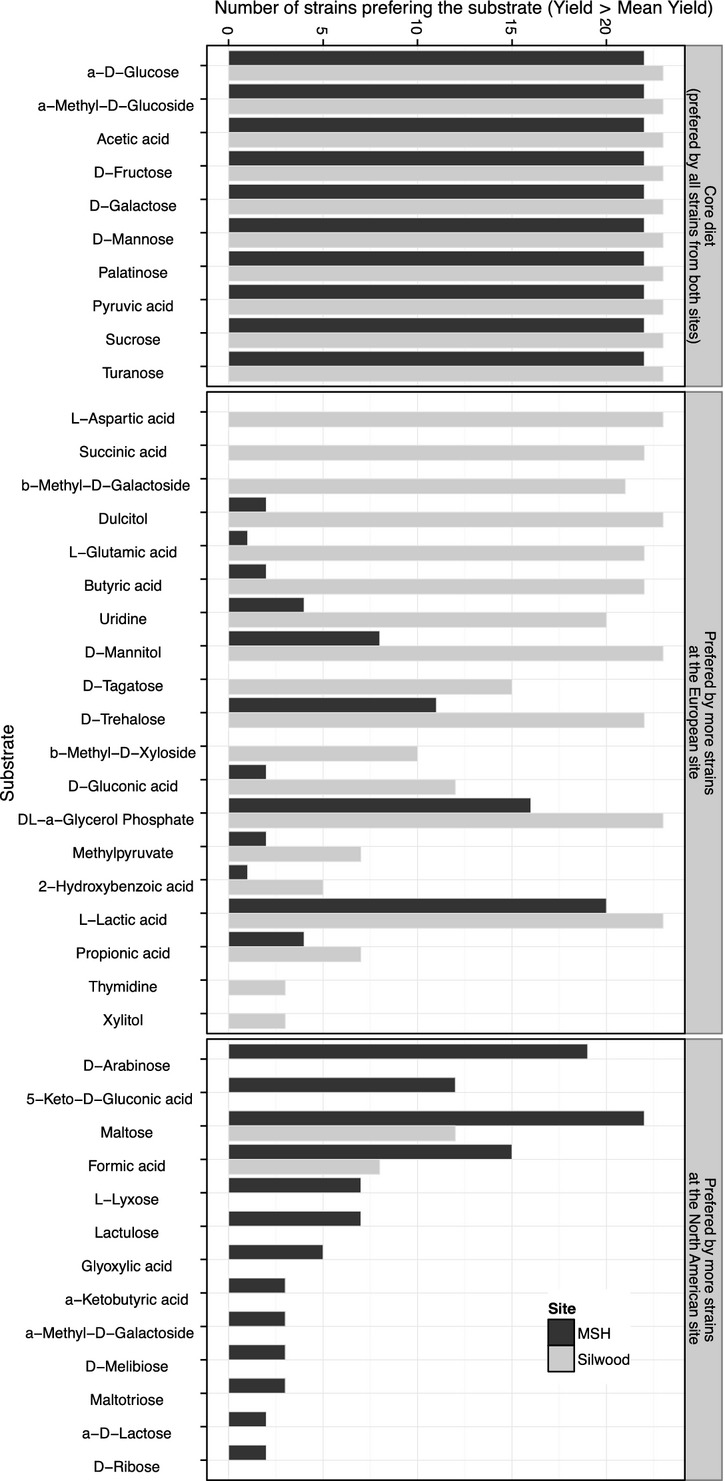
Number of isolates from MSH (black) and Silwood (gray) preferring given substrates. The criterion for preference is that the yield deviation from mean yield across all substrates is positive, that is, yield on target substrate > mean yield across substrates. Substrates in the core diet are preferred by all isolates from both locations. Substrates from the ancillary diet that differ in preference between sites are grouped according to the site with most isolates preferring the substrate and ordered by the difference in isolate preference between sites.

There are other substrates that were preferred (higher than average standardized yield on these substrates) by many isolates at one locality but by few or none at the other (Fig.6). Most Silwood isolates preferred substrates that are metabolized through the alanine, aspartate, and glutamate pathway (l-aspartic acid and b-methyl-d-galactoside; some isolates could also utilize d-alanine) and through the galactose pathway (dulcitol (galactitol), d-tagatose, and stachyose), while few if any of the MSH isolates could metabolize these substrates.

### Regional and local dietary specialization

Within each site, the geographical distance between isolates explains part of the genetic variance in the pattern of substrate utilization (Mantel test, *r* = 0.16, *P* = 0.02 for European site, and *r* = 0.28, *P* = 0.002 for the North American site; see Legendre and Fortin 2010). Despite this geographic variation in substrate utilization, patterns of utilization of substrates were not sufficiently different to allow the differentiation between isolates (0% consistent proper classification when using classification algorithms), and no consistent pattern between sites was evident either among the substrates that varied most between isolates at each location or among the substrates that varied most with latitude and longitude.

The MSH sample includes isolates from two major geographical races that are found in different areas of the mountain. These clades also display different patterns of substrate use (Fig.5).

## Discussion

### Breadth and variation of diet

Most of the efficiently utilized substrates in the core diet are sugars. The ancillary diet is quite extensive, however, including about half the substrates tested. Wild yeast is able to grow, albeit inefficiently, on the random mixtures of substrates which it is likely to encounter in natural environments. Our surveys confirm that there is genetic variation in metabolic capacity within natural populations of a eukaryotic microbe. Isolates varied consistently in the amount of metabolic activity for several substrates, and isolates from the two sites could be distinguished on the basis of their pattern of substrate utilization.

### Geographical variation in substrate utilization

There is geographical variation of substrate use in *S. paradoxus* at continental, regional, and local scales. The divergence of substrate utilization between North American and European isolates is associated with the genetic divergence of European and North American populations documented by previous genetic surveys of isolates from MSH, Silwood, and other sites (Koufopanou et al. 2006). In a similar fashion, wild *Saccharomyces cerevisiae* ecotypes have specialized on vineyards and oaks separately, with oak ecotypes found within vineyards but not vice versa (Hyma and Fay 2013). In *S. paradoxus*, this divergence has gone some way toward speciation; the European and North American populations were in fact distinguished as *S. paradoxus* and *Saccharomyces cariocanus* until recently. The MSH site happens to straddle the boundary between two North American races of *S. paradoxus*, and isolates can be assigned to one or the other by genetic criteria (Leducq et al. 2014). Moreover, it has been recently shown that these two races are partially reproductively isolated (Charron et al. 2014). As with continental variation, regional genetic divergence is associated with consistent differences in the pattern of substrate use. Finally, there is evidence for spatial variation of substrate use at both localities on scales of tens or hundreds of meters.

### Purifying and diversifying selection for substrate utilization

Genetic variation for substrate utilization may be maintained through the balance between deleterious mutation and purifying selection or through diversifying selection arising either from functional interference in substrate utilization or from mutational degradation of unused metabolic pathways.

First, the local variation in metabolic performance that we have found might be attributable in part to the balance between mutation and purifying selection. This seems quantitatively plausible because the average amount of genetic variation in yield is comparable with the amount of genetic variation in fitness estimated in natural populations of other kinds of organism. The advance in fitness per generation, by the fundamental theorem of natural selection, is equal to the standardized variance of fitness (SV_A_, additive variance divided by the square of the mean). This is the square of the genetic coefficient of variation, which has an average value of SV_A_ = 0.04 at MSH and 0.02 at Silwood. An experimental study of fitness in a natural population of the annual herb *Impatiens pallida* at Mont St-Hilaire gave an estimate of SV_A_ = 0.03 (Schoen et al. 1994). Other studies of birds and plants suggest that SV_A_ is usually in the region of 0.01–0.1 in natural conditions of growth (Bell 2008).

An alternative explanation for the maintenance of diversity is that variation is protected by diversifying selection. The ability to use the whole range of available substrates may be constrained by intrinsic physiological trade-offs, functional interference, among different kinds of substrates, such that the enhanced ability to utilize some is necessarily accompanied by reduced ability to utilize others. Alternatively, if some substrates are lacking for a long period of time, the ability to consume them is not maintained by selection. Mutations in the genes that govern consumption will then be neutral, and will tend to accumulate over time. Variation in substrate availability over sites will then lead to a spatial pattern of specialization. Such trade-offs are evaluated through the genetic (among-isolate) correlations between substrates or kinds of substrate. The prevalence of positive correlation caused by overall genetic variance is not necessarily inconsistent with an underlying tendency for correlations to be negative for isolates with equivalent overall performance.

We found two trade-offs that we were able to identify with confidence because they involve consistent negative correlations between substrates in broad predefined categories. One involves the interference between substrates in the core diet and those in the ancillary diet, while the other involves the two main types of sugars, hexoses, and pentoses. This might in principle lead to the divergence of two or more ecotypes. The trade-off between pentose and hexose utilization, indeed, appears to be one of the drivers of divergence between the Silwood population, which may have lost some of its pentose utilization function, and the MSH population, which may have specialized on pentose utilization. In laboratory strains of *Saccharomyces cerevisiae* bearing mutations in the pentose phosphate pathway, growth on hexoses is not compromised (Lobo and Maitra 1982), while growth on gluconate has led to the isolation of strains with increased ability to utilize gluconate at the expense of their ability to grow on glucose (Cadière et al. 2011).

To distinguish between purifying and diversifying selection as explanations of variation, the crucial prediction is that substrate use by isolates will correspond with substrate availability at sites if variation is adaptive. Opulente et al. (2013) showed that metabolic patterns in 448 strains of the genus *Saccharomyces* were partially predictable from the environments where the strains occurred. Further progress in understanding the maintenance of metabolic variation in microbial populations is likely to depend on elucidating the availability of substrates in natural habitats and relating it to the metabolic specialization of resident strains.

## Supporting Information

**Figure S1.** Growth of PM1 and PM2A plates that were inoculated with cell mixture supplemented with and without tetrazolium were highly correlated after 72 h of growth (*R*^2^ = 0.7556).

**Table S1.** GPS coordinates of MSH trees.

**Table S2.** GPS coordinates of Silwood trees.

**Data S1.** MSH data.

**Data S2.** PM1 and PM2 dye and no dye correlation data

**Data S3.** R scripts.

**Data S4.** Silwood Data.
