# Supplementary figures and images for: Metabolic variation in natural populations of wild yeast
Source: Ecol Evol. 2015 Jan 14;5(3):722–32. doi: 10.1002/ece3.1376 (PMC4328774; doi:10.1002/ece3.1376)

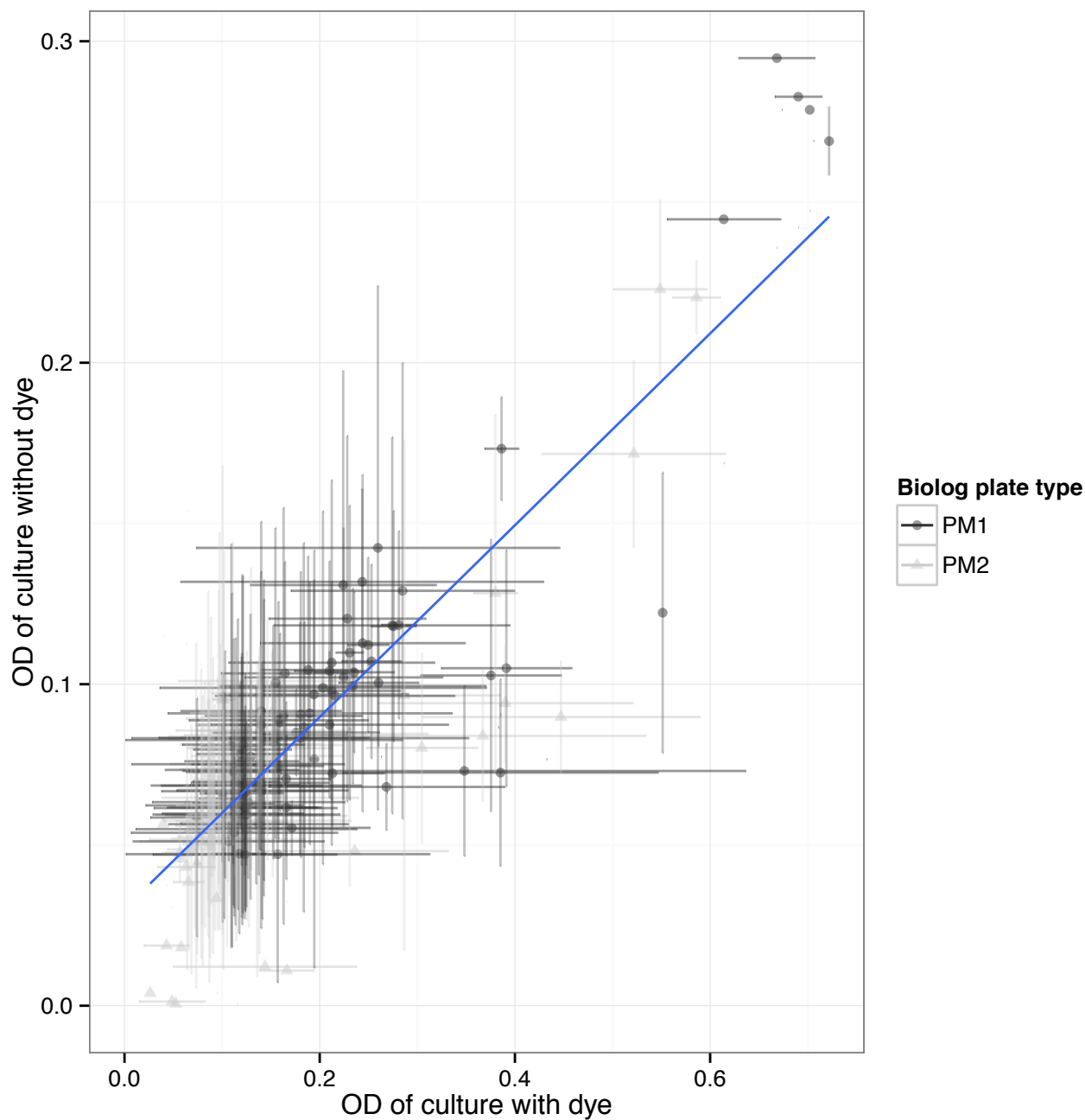

Supplement: Supplementary file 1 [file ece30005-0722-sd1.pdf]
