# Supplementary material for: Metabolic variation in natural populations of wild yeast
Source: Ecol Evol. 2015 Jan 14;5(3):722–32. doi: 10.1002/ece3.1376 (PMC4328774; doi:10.1002/ece3.1376)
Supplement: Supplementary file 2 [file ece30005-0722-sd2.pdf]

site	Strain #	tree #	latitude	longitude	elevation (m)
Burned hill	139	3004	45°32'29.58"N	73°9'55.20"W	252
Burned hill	148	3006	45°32'29.65"N	73°9'54.36"W	265
Burned hill	604	3012	45°32'29.11"N	73°9'53.25"W	259
Dieppe	23	567	45°33'40.25"N	73°10'28.42"W	363
Dieppe	44	826	45°33'39.82"N	73°10'29.85"W	350
Dieppe	D1S11	not labeled	45°33'40.54"N	73°10'29.76"E	-
Dieppe	D2S35	823	45°33'40.43"N	73°10'29.24"W	-
Dieppe	D2B12	822	45°33'40.29"N	73°10'28.35"W	-
East hill	620	850	45°32'46.2"N	73°08'34.2"W	274
Lake hill	475	846	45°32'22.8"N	73°08'59.8"W	258.2
Lake hill	483	3070	45°32'09.0"N	73°08'55.2"W	127.4
Lake hill	498	3077	45°32'20.1"N	73°08'57.0"W	269.8
Lake hill	544	839	45°32'17.1"N	73°08'55.8"W	231.1
Nature center	95	578	45°32'22.30"N	73°9'23.49"W	193
Nature center	503	830	45°32'21.55"N	73°9'23.10"W	176
Nature center	584	575	45°32'21.03"N	73°9'23.64"W	177.6
Pain de Sucre	19	587	45°33'2.48"N	73°10'19.47"W	406
Pain de Sucre	20	588	45°33'2.61"N	73°10'19.70"W	415
Rocky	134	3052	45°33'28.39"N	73°10'8.56"W	348
Rocky	147	3061	45°33'19.92"N	73°09'48.99"W	401
Rocky	169	3056	45°33'29.53"N	73°10'6.11"W	350
Rocky	605	3052	45°33'28.39"N	73°10'8.56"W	348
