# Supplementary material for: Metabolic variation in natural populations of wild yeast
Source: Ecol Evol. 2015 Jan 14;5(3):722–32. doi: 10.1002/ece3.1376 (PMC4328774; doi:10.1002/ece3.1376)
Supplement: Supplementary file 3 [file ece30005-0722-sd3.pdf]

| region | site | tree | latitude | longitude | elevation (m) |
|---|---|---|---|---|---|
| Windsore park | A | Q14.4 | 51°25'15.40"N | 0°38'15.70"W | 55 |
| Windsore park | A | Q15.1 | 51°25'15.26"N | 0°38'11.40"W | 54 |
| Windsore park | B | Q31.4 | 51°24'40.56"N | 0°37'7.18"W | 46 |
| Windsore park | B | Q32.3 | 51°24'39.70"N | 0°37'6.00"W | 46 |
| Windsore park | A | Q4.1 | 51°25'12.87"N | 0°38'16.35"W | 56 |
| Windsore park | B | Q43.5 | 51°24'39.83"N | 0°37'10.54"W | 46 |
| Windsore park | C | Q59.1 | 51°24'54.65"N | 0°37'35.56"W | 58 |
| Windsore park | A | Q6.1 | 51°25'11.25"N | 0°38'13.56"W | 60 |
| Windsore park | C | Q62.5 | 51°24'54.74"N | 0°37'29.69"W | 58 |
| Windsore park | C | Q69.8 | 51°24'57.59"N | 0°37'34.64"W | 64 |
| Windsore park | C | Q74.4 | 51°24'58.02"N | 0°37'12.67"W | 73 |
| Windsore park | C | Q95.3 | 51°24'57.88"N | 0°37'16.46"W | 74 |
| Windsore park | C | Q89.8 | 51°25'0.45"N | 0°37'13.11"W | 75 |
| Silwood | D | S36.7 | 51°24'34.82"N | 0°38'35.71"W | 66 |
| Silwood | D | T18.2 | 51°24'40.68"N | 0°38'39.42"W | 64 |
| Silwood | D | T21.4 | 51°24'39.43"N | 0°38'42.48"W | 66 |
| Silwood | B | T26.3 | 51°24'28.32"N | 0°38'39.37"W | 68 |
| Silwood | D | T32.1 | 51°24'37.01"N | 0°38'36.11"W | 62 |
| Silwood | F | T62.1 | 51°24'51.30"N | 0°39'15.87"W | 67 |
| Silwood | F | T68.2 | 51°24'53.80"N | 0°39'9.09"W | 66 |
| Silwood | C | T76 (T76.6 | 51°24'44.70"N | 0°38'29.13"W | 62 |
| Silwood | A | T8.1 | 51°24'25.59"N | 0°38'49.64"W | 61 |
| Silwood | B | W7 | 51°24'29.01"N | 0°38'36.74"W | 69 |
| Silwood | C | Y6.5 | 51°24'35.85"N | 0°38'21.69"W | 69 |
| Silwood | C | Y7 | 51°24'35.85"N | 0°38'21.69"W | 69 |
